# Increasing obesity and comorbidity in patients undergoing primary total hip arthroplasty in the U.S.: A 13-year study of time trends

**DOI:** 10.1186/1471-2474-15-441

**Published:** 2014-12-17

**Authors:** Jasvinder A Singh, David G Lewallen

**Affiliations:** Medicine Service, Birmingham VA Medical Center, Birmingham, AL USA; Department of Medicine at School of Medicine, and Division of Epidemiology at School of Public Health, University of Alabama, Faculty Office Tower 805B, 510 20th Street S, Birmingham, AL 35294 USA; Department of Orthopedic Surgery, Mayo Clinic College of Medicine, Rochester, MN USA

**Keywords:** Total hip replacement, Time trends, Arthroplasty, Joint replacement, Diagnosis, Obesity, Comorbidity, Osteoarthritis

## Abstract

**Background:**

Few, if any data are available are available regarding the time-trends in characteristics of patients who have undergone primary THA. Our objective was to examine the time-trends in key demographic and clinical characteristics of patients undergoing primary total hip arthroplasty (THA).

**Methods:**

We used the data from the Mayo Clinic Total Joint Registry from 1993–2005 to examine the time-trends in demographics (age, body mass index (BMI)), medical (Deyo-Charlson index) and psychological comorbidity (anxiety, depression) and underlying diagnosis of patients undergoing primary THA. Chi-square test and analysis for variance were used. Multivariable-adjusted logistic regression (age, sex, comorbidity-adjusted) compared 1993–95 to other study periods. Odds ratio (OR) and 95% confidence interval (CI) are presented.

**Results:**

The primary THA cohort consisted of 6,168 patients with 52% women. In unadjusted analyses, compared to 1993–95, significantly more patients (by >2-times for most) in 2002–05 had: BMI ≥ 40, 2.3% vs. 6.3%; depression, 4.1% vs. 9.8%; and anxiety, 3.4% vs. 5.7%; and significantly fewer had an underlying diagnosis of rheumatoid/inflammatory arthritis, 3.7% vs. 1.5% (p ≤ 0.01 for all). In multivariable-adjusted models, compared to 1993–95, significantly more patients in 2003–05 had (all p-values ≤ 0.01): BMI ≥ 40, OR, 2.79 (95% CI: 1.85, 4.22); Deyo-Charlson Index ≥ 3, 1.32 (1.07, 1.63); depression, 2.25 (1.66, 3.05); and anxiety, 1.71 (1.19, 2.15). Respectively, fewer patients had a diagnosis of RA/inflammatory arthritis: 0.28 (0.17, 0.46; p < 0.01). Over the 13-year study period, Deyo-Charlson index increased by 22% (0.9 to 1.1) and the mean age decreased by 0.7 years (65.0 to 64.3) (p < 0.01 for both).

**Conclusions:**

Obesity, medical and psychological comorbidity increased and the underlying diagnosis of RA/inflammatory arthritis decreased rapidly in primary THA patients over 13-years. Our cohort characteristics are similar to previously described characteristics of national U.S. cohort, suggesting that these trends may be national rather than local trends. This is important information for policy makers to take into account for resource allocation. Studies of THA outcomes and utilization should take these rapidly changing patient characteristics into account.

## Background

Total hip arthroplasty (THA) has been called the operation of the century [1]. Primary THA is the second most common joint replacement surgery performed in the U.S. [2], associated with a significant improvement in patient outcomes [3]. The utilization of primary THA is rapidly increasing and 478,000 primary THA procedures were performed in the U.S. in 2009 [4, 5]. Recent studies have described the time-trends in THA utilization [6–10], outcomes [6, 8–11] and select patient characteristics (age, obesity) [6, 9, 10]. On the other hand, time-trends in important characteristics of patients undergoing THA, including body mass index (BMI), medical/ psychological comorbidity etc. associated with THA outcomes, have not been examined. Most studies were done in non-U.S. settings [8, 11], except three key studies using data from the Medicare [6], the National Inpatient sample (NIS) [9] and the National Hospital Discharge Survey (NHDS) [10]. Several knowledge gaps exist in this area, as summarized in the following section.

In Medicare recipients 65 years and older, the number of comorbidities increased from 1.0 to 2.0 and obesity rates increased from 2.2% to 7.6% from 1991 to 2008 [6]. Representative studies of the U.S. NIS from 1998 to 2008 and U.S. NHDS data 1990 to 2004, that included all age groups, showed that among patients undergoing THA, age decreased by 2.1 years [9], Deyo comorbidity index increased from 0.48 to 0.65 [9] and obesity increased from 2.2% to 3.8% [10]. Data from these Medicare, NIS and NHDS studies are limited in several important aspects: (1) obesity, linked to THA outcomes [12–15], was captured with a diagnostic code, which is associated with significant under-coding compared to when using the weight and height data [16], as evident in low obesity rates <5% in these studies vs. U.S. obesity rates of 37% in 2009, proving that the use of ICD-9 code for obesity leads to high misclassification rate [17]; and (2) anxiety, depression and underlying diagnosis, important predictors of THA outcomes [14, 18–22], were not assessed. Data from Medicare study are additionally limited in that they are not representative, since at least 1/3rd of all THAs are performed in those younger than 65 years [23, 24], the fastest growing age group for the receipt of THA over time [25]. A recent study in TKA cohort found that rates of extreme obesity, Deyo-Charlson index score > =3, depression and anxiety increased 2–3 fold from 1993–2005 [26]. To our knowledge, there is lack of such data for time-trends in BMI, anxiety, depression and underlying diagnoses in patients undergoing THA in the U.S.

One of the most remarkable changes in the epidemiology of primary THA in the last two decades is the expanding indication of THA to both younger and older patients. Studies have not examined whether the time-trends in important clinical characteristics (BMI, underlying diagnosis and medical and psychiatric comorbidity) vary by age, the most rapidly changing characteristic of patients undergoing primary THA. Knowing how the patient complexity has changed over time is critically important, since these patient characteristics can have a significant impact on THA outcomes. Our objectives were to: (1) examine the time trends in BMI, underlying diagnosis and medical and psychiatric comorbidity in patients undergoing primary THA; and (2) assess whether these time-trends are different in various patient age groups.

## Methods

We report the methods and results as recommended in the Strengthening of Reporting in Observational studies in Epidemiology (STROBE) statement [27].

### Study cohort selection

The Mayo Clinic Total Joint Registry, a large institutional registry that has prospectively collected preoperative and postoperative follow-up data on all THA patients at the Mayo Clinic, Rochester, served as the data source for this study [28]. Preoperative data, the focus of this study, are collected at the in-clinic visit by the orthopedic surgeons using a standardized data collection form. Since 1993, trained, dedicated Total Joint Registry staff has captured and entered these data into electronic database and ensured the completeness of data. The Mayo Clinic’s Institutional Review Board approved the research study. All investigations were conducted in conformity with ethical principles of research.

### Study outcomes

The study period was divided into roughly equal four intervals (1993–95, 1996–98, 1999–2001 and 2002–05). The study outcomes of interest were patient’s demographic and clinical characteristics and medical and psychological comorbidity. Patient characteristics and clinical variables were categorized as follows. BMI was categorized as <25, overweight, 25–29.9, obese and very obese (obesity class I and II), 30–39.9, or extremely obese (obesity class III), ≥40 as previously [14, 29, 30], as per WHO classification [31]. Age was categorized as <50, 50- < 65, 65- < 80, ≥80, to allow comparison to other studies using Medicare data that only include patients 65 years and older. The operative diagnosis was categorized as osteoarthritis, rheumatoid arthritis (RA)/inflammatory arthritis and other.

Medical comorbidity was assessed with the Deyo-Charlson index [32], a validated measure of comorbidity. It is a summative weighted score based on the presence of 17 comorbidities (including cardiac, pulmonary, renal, hepatic disease, diabetes, cancer, hemiplegia etc.) [33, 34], captured based on the presence of International Classification of Diseases, ninth revision, common modification (ICD-9-CM) diagnostic codes. Deyo-Charlson index was categorized as none, one, two and three or more, similar to previous studies [35], and comorbidities were also assessed individually. Depression and anxiety were assessed based on the presence of ICD-9-CM diagnostic codes.

### Statistical analyses

We calculated summary statistics as proportion or mean (standard deviation). We used chi-square tests to compare the proportions of patients with each category of patient characteristic (BMI, underlying diagnosis, Deyo-Charlson index, depression and anxiety) over time, and also also comparisons of categorical variables in 1993–95 vs. 2005–08. We used the analysis of variance to compare means (age, BMI, Deyo-Charlson index) in various time periods.

We also examined the time trends in the proportion of patients with extreme obesity (BMI ≥40; obesity class III) and high comorbidity (Deyo-Charlson index ≥3) using logistic regression. We examined the time trends in BMI ≥40, RA/inflammatory arthritis as underlying diagnosis, Deyo-Charlson index of ≥3, depression and anxiety, within each age group, using multivariable-adjusted logistic regression analyses. Time-period was considered in four category nominal variable (1993–1995, reference category), and the BMI <40, other diagnoses, Deyo-Charlson index of ≤2 and absence of depression or anxiety serving as the reference category for each respective outcome. The overall p-value for the variable was considered as an indicator of whether there was a period effect; visual inspection of the odds ratios was interpreted for time-trend. We performed statistical analyses using Statistical Package for Social Sciences (SPSS, version 21, Chicago, IL). A p-value <0.05 was considered significant.

## Results

The primary THA cohort consisted or 6,168 patients, 52% were women, 10% were 80 years or older, 5% had BMI of 40 or more, 38% had ASA grade III-IV, the underlying diagnosis was OA in 87% and 64% had cemented/hybrid implant fixation (Table 1).

**Table 1:** **Preoperative patient characteristics of the primary THA cohort**

	Primary THA (n = 6,168) N (%) or Mean (SD)
Gender	
Females	3175 (52%)
Males	2993 (48%)
Age	
<50	975 (16%)
50- < 65	1626 (26%)
65- <80	2947 (48%)
≥80	620 (10%)
Body Mass Index (BMI)	
<25	1505 (24%)
25**–** 29.99	2356 (38%)
30**–** 34.99	1497 (24%)
35**–** 39.99	506 (8%)
≥40	279 (5%)
American Society of Anesthesiologists (ASA) class	
I	359 (6%)
II	3468 (56%)
III	2256 (37%)
IV	59 (1%)
Underlying Diagnosis	
Inflammatory arthritis	162 (2%)
Osteoarthritis	5339 (87%)
Other	666 (11%)
Cement Fixation	
Uncemented	2224 (36%)
Cemented/hybrid	3943 (64%)
Age, mean (SD)	64.2 (13.8)
Deyo-Charlson Index, mean (SD)	1.0 (1.9)
BMI, mean (SD)	28.9 (5.8)

Missing data: BMI, n = 25; ASA score, n = 26; Underlying diagnosis, n = 1.

### Time trends in socio-demographic and clinical characteristics and comorbidity

The mean age decreased by 0.7 years and mean BMI increased by 1.6 kg/m^2^ over the 13-year period of study, both statistically significant. The prevalence of BMI ≥30 (obesity class I-III) and ≥40 (obesity class III) increased from 33.2% to 37.1% and 2.3% to 6.3% during the study period, respectively. The proportion of younger patients (≤50 years) increased from 13.7% to 16% and the prevalence of RA/inflammatory arthritis as the underlying diagnosis decreased from 3.7% in 1993–95 to 1.3% in 2002–05 (Table 2). The proportion of primary THA patients with Deyo-Charlson score of 3 or more increased from 12.2% to 15.1%. The prevalence of psychological comorbidity increased over the study period: depression increased >2-fold from 4.5% to 9.8% and anxiety from 3.4% to 5.7% (Table 2).

**Table 2:** **Time trends in demographics and clinical characteristics of patients undergoing primary THA, in %, unless specified otherwise**

	1993-1995	1996-1998	1999-2001	2002-2005	P-value
Age category					**0.002**
<50	13.7	17.1	16.2	16.0	
50- < 65	24.7	25.9	26.6	27.5	
65- <80	51.8	48.8	47.2	44.9	
≥80	9.8	8.2	10.0	11.6	
Mean age (SD)	65.0 (13.2)	63.5 (14.0)	63.9 (14.0)	64.3 (13.9)	**0.03**
Female gender	51.8	50.0	52.0	51.9	0.67
BMI					**<0.001**
<25	28.1	26.0	24.0	21.5	
25**–** 29.9	38.8	40.0	37.9	37.2	
30**–** 34.9	23.5	24.1	23.6	25.8	
35**–** 39.9	7.3	6.6	9.1	9.3	
≥40	2.3	3.3	5.3	6.3	
Mean BMI (SD)	28.0 (5.2)	28.3 (5.2)	29.1 (6.0)	29.6 (6.2)	**<0.001**
Operative Diagnosis					**<0.001**
Osteoarthritis	81.8	81.0	88.2	92.2	
RA/inflammatory arthritis	3.7	3.7	2.4	1.3	
Other	14.5	15.3	9.4	6.5	
Deyo-Charlson Index					**<0.001**
0	61.9	63.2	59.2	56.7	
1	17.3	17.2	15.4	16.2	
2	8.7	9.3	11.0	11.9	
3 or more	12.2	10.2	14.4	15.1	
Depression	4.5	4.8	7.2	9.8	**<0.001**
Anxiety	3.4	3.5	4.9	5.7	**0.003**

SD, standard deviation; RA, rheumatoid arthritis, BMI, body mass index.

Significant p-values are in bold. We used analysis of variance for continuous variables and, chi-squared test for categorical variables.

Compared to 1993–95, significantly more patients (by >2-times for most) in 2002–05 had: BMI ≥ 40, 2.3% vs. 6.3%; depression, 4.1% vs. 9.8%; and anxiety, 3.4% vs. 5.7%; and significantly fewer had an underlying diagnosis of rheumatoid/inflammatory arthritis, 3.7% vs. 1.5% (p ≤ 0.01 for all comparisons using chi-square tests).

The prevalence for several Deyo-Charlson comorbidities increased significantly over the study period and the mean Deyo-Charlson comorbidity index increased by 22% (Table 3).

**Table 3:** **Time trends in prevalence of medical comorbidity (in %) from 1993 to 2005 in patients undergoing primary THA**

	1993-1995	1996-1998	1999-2001	2002-2005	P-value
Deyo-Charlson comorbidities					
Myocardial Infarction	4.0	4.2	4.1	4.7	0.76
Congestive Heart Failure	2.5	2.8	3.5	4.1	0.05
Peripheral Vascular Disease	4.7	3.8	4.2	5.6	0.07
Cerebrovascular disease	5.5	5.2	6.3	7.9	**0.006**
Dementia	0	0	0.3	0.4	**0.02**
COPD	9.7	8.8	10.2	9.7	0.66
Peptic ulcer disease	5.5	6.3	6.2	6.3	0.81
Mild liver disease	1.4	0.6	1.1	1.2	0.20
Diabetes	4.8	5.0	5.9	8.0	**<0.001**
Diabetes with end organ damage	0.9	0.7	1.8	2.0	**0.005**
Hemiplegia	0.3	0.2	0.2	0.5	0.48
Moderate/severe renal disease	2.6	3.2	5.4	5.9	**<0.001**
Moderate/severe liver disease	0.5	0.3	0.2	0.3	0.58
Metastatic solid tumor	3.3	2.3	3.6	2.7	0.12
AIDS	0	0.1	0	0	0.32
Rheumatologic disease	6.1	5.2	5.4	5.2	0.67
Other cancer	11.2	10.9	13.5	13.9	**0.02**
**Mean Deyo-Charlson score (SD)**	0.9 (1.8)	0.9 (1.7)	1.1 (2.0)	1.1 (2.0)	**<0.001**

COPD, Chronic Obstructive Pulmonary Disease; AIDS, Acquired Immune Deficiency Disease; SD, standard deviation.

Significant p-values are in bold, calculated with chi-squared test.

### Time trends in key patient characteristics within each age group

We noted a significant increase in prevalence of BMI ≥40 (>2-fold increase each) in two age groups, 50- < 65 and 65- < 80 (Table 4). Similarly, a significant decline in the underlying diagnosis of RA/inflammatory arthritis was noted in the two age groups, those <50 years from 17.9% to 4.2% (77% reduction) and in those 65- < 80 years from 3.5% to 1.2% (66% reduction). The proportion of patients with Deyo-Charlson index of 3 or more increased in 65- < 80 years group, from 16.1% to 19.9% (23% increase; Table 4). The prevalence of depression increased significantly across all four age groups, most impressive in patients younger than 50 years, from 2.9% to 8.3% (almost 3-fold). The prevalence of anxiety increased in all age groups, but was not statistically significant, except for a trend in 80 and older age group, from 3.4% to 5.7% (p = 0.06; Table 4).

**Table 4:** **Time-trends in patient characteristics (in %) by patient age category from 1993 to 2005 in patients undergoing primary THA**

	1993-1995	1996-1998	1999-2001	2002-2005	P-value
BMI ≥40					
Age, <50	1.1	7.3	7.2	5.8	0.30
Age, 50- < 65	3.5	5.3	7.1	9.3	**0.007**
Age, 65- <80	2.4	1.3	4.6	5.7	**<0.001**
Age ≥80	0	0	1.3	2.2	0.17
Diagnosis of RA/inflammatory arthritis					
Age, <50	17.9	10.5	6.2	4.2	**<0.001**
Age, 50- < 65	2.6	3.3	1.9	1.0	0.11
Age, 65- <80	3.5	4.2	2.3	1.2	**0.002**
Age ≥80	0	0	1.4	0	0.11
Deyo-Charlson Index ≥3					
Age, <50	5.7	1.7	4.8	4.8	0.16
Age, 50- < 65	4.1	6.7	7.8	8.2	0.13
Age, 65- <80	16.1	12.7	19.8	19.9	**<0.001**
Age ≥80	20.8	24.1	21.9	27.2	0.51
Depression					
Age, <50	2.9	3.0	6.0	8.3	**0.02**
Age, 50- < 65	5.4	4.8	8.7	9.4	**0.02**
Age, 65- <80	4.7	5.2	6.8	9.1	**0.002**
Age ≥80	4.0	6.3	7.1	15.8	**0.001**
Anxiety					
Age, <50	1.7	0.9	2.4	2.9	0.41
Age, 50- < 65	2.2	2.2	4.9	3.9	0.12
Age, 65- <80	4.1	5.1	4.8	6.6	0.14
Age ≥80	3.4	3.5	4.9	5.7	0.06

Significant p-values are in bold, calculated with chi-squared test.

### Unadjusted and adjusted odds in key patient characteristics over time

In the unadjusted analyses, compared to 1993–95, the odds of BMI ≥40 in the next three time periods were 1.46, 2.43 and 2.90 (Table 5). These odds attenuated minimally after adjusting for age-, sex- and comorbidity in the multivariable-adjusted models (Table 5). Compared to 1993–95, the unadjusted odds of RA/inflammatory arthritis as the underlying diagnosis were 1.00, 0.59 and 0.31 in the three next time periods (Table 5). The respective unadjusted odds for medical and psychological comorbidity were as follows: (1) for Deyo-Charlson index of 3 or more, 0.82, 1.21 and 1.28; (2) depression, 1.07, 1.64 and 2.30; and (3) anxiety, 1.04, 1.48 and 1.74, respectively (Table 5). The higher odds for Deyo-Charlson, depression and anxiety also were minimally attenuated with the adjustment for age, sex- and comorbidity in the multivariable-adjusted models (Table 5).

**Table 5:** **Unadjusted and adjusted odds of extreme obesity (BMI of 40 or higher), an underlying operative diagnosis of RA/inflammatory arthritis, Deyo-Charlson index of 3 or higher, depression or anxiety over time**

	1993-1995	1996-1998	1999-2001	2002-2005
**BMI ≥40**				
Unadjusted	Ref	1.46 (0.91, 2.35)	2.43 (1.58, 3.73)*	2.90 (1.92, 4.37)*
Age- and sex-adjusted	Ref	1.41 (0.85, 2.26)	2.36 (1.53, 3.63)*	2.84 (1.88, 4.29)*
Comorbidity-adjusted	Ref	1.47 (0.92, 2.37)	2.40 (1.56, 3.69*	2.87 (1.90, 4.33)*
Age-, sex- and comorbidity adjusted	Ref	1.42 (0.88, 2.28)	2.30 (1.50, 3.55)*	2.79 (1.85, 4.22)*
**Diagnosis of RA/inflammatory arthritis** ^**a**^				
Unadjusted	Ref	1.00 (0.67, 1.50)	**0.59 (0.38, 0.91)** ^**¶**^	0.31 (0.19, 0.51)*
Age- and sex-adjusted	Ref	0.97 (0.65, 1.46)	**0.55 (0.35, 0.85)** ^**†**^	0.29 (0.18, 0.48)*
Comorbidity-adjusted	Ref	1.00 (0.67, 1.51)	**0.58 (0.38, 0.90)** ^**¶**^	0.31 (0.19, 0.50)*
Age-, sex- and comorbidity adjusted	Ref	0.99 (0.66, 1.48)	**0.53 (0.34, 0.83)** ^**†**^	0.28 (0.17, 0.46)*
**Deyo-Charlson Index ≥3** ^**b**^				
Unadjusted	Ref	0.82 (0.64, 1.04)	1.21 (0.97, 1.51)	**1.28 (1.04, 1.58)** ^**¶**^
Age- and sex-adjusted	Ref	0.86 (0.67, 1.10)	**1.26 (1.01, 1.58)** ^**¶**^	**1.32 (1.07, 1.63)** ^**†**^
**Depression**				
Unadjusted	Ref	1.07 (0.74, 1.53)	**1.64 (1.18, 2.27)** ^**†**^	2.30 (1.70, 3.11)*
Age- and sex-adjusted	Ref	1.09 (0.76, 1.56)	**1.65 (1.19, 2.29)** ^**†**^	2.31 (1.71, 3.13)*
Comorbidity-adjusted	Ref	1.09 (0.76, 1.56)	**1.59 (1.15, 2.21)** ^**†**^	2.24 (1.66, 3.04)*
Age-, sex- and comorbidity adjusted	Ref	1.11 (0.77, 1.59)	**1.60 (1.15, 2.22)** ^**†**^	2.25 (1.66, 3.05)*
**Anxiety**				
Unadjusted	Ref	1.04 (0.69, 1.59)	**1.48 (1.01, 2.17)** ^**¶**^	**1.74 (1.22, 2.49)** ^**†**^
Age- and sex-adjusted	Ref	1.09 (0.72, 1.66)	**1.51 (1.03, 2.21)** ^**¶**^	**1.75 (1.22, 2.51)** ^**†**^
Comorbidity-adjusted	Ref	1.07 (0.70, 1.62)	1.44 (0.98, 2.11)	**1.70 (1.18, 2.43)** ^**†**^
Age-, sex- and comorbidity adjusted	Ref	1.10 (0.73, 1.68)	1.47 (1.00, 2.15)	**1.71 (1.19, 2.15)** ^**†**^

Ref, reference category, has an odds ratio of 1.0.

^a^Osteoarthritis is the reference category for diagnosis.

^b^Since the variable under study was Deyo-Charlson index, comorbidity-adjusted models were not run.

^¶^P < 0.05; ^†^P ≤ 0.01; *P ≤ 0.001.

Significant odds ratios are in bold.

## Discussion

In this study in patients undergoing primary THA, we found increasing rates of obesity, medical and psychological comorbidity and a decreasing rate of RA/inflammatory arthritis as the underlying diagnosis over time. To our knowledge, this is the first study to comprehensively examine time-trends in depression, anxiety, medical comorbidities and underlying diagnosis, overall and in different age groups of patients undergoing primary THA. The younger age group is the fastest growing group for the receipt of THA over time among all age groups [23, 25]. Several study findings deserve further discussion.

A key finding from our study was that the prevalence of obesity, including extreme obesity (BMI ≥40), differed significantly by time and seemed to increase over time. By using the height and weight data prospectively captured in the joint registry, we were able to get accurate estimates of obesity and overcame a key limitation of under-diagnosis of obesity based on the use of ICD-9-CM diagnostic code [16] in all the previous studies [6, 10]. Among the patients undergoing primary THA, obesity increased from 33.2% in 1992–95 to 37.1% for in 2002–05. In the U.S. Medicare population, the prevalence of obesity was reported to increase from 2.2% in 1991 to 7.6% in 2008 [6] and in the NIS from 2.2% in 1990 to 5.0% in 2004 [10]. Thus, our study is the first to examine the change in BMI over time in a U.S. cohort undergoing THA. The prevalence of obesity of 37% in our 2002–2005 cohort was higher than the 24.5% reported in 2004 for the general U.S. population [17]. Comparing the prevalence of obesity of 5% in the NIS study using the ICD-9 code to the 37% using height and weight data in our study, highlights the extent of under-coding of obesity using ICD-9-CM codes in administrative databases (by 7-fold relative and 32% absolute difference) and the resulting misclassification in earlier studies. Our cohort's demographic and clinical characteristics are similar to those reported from the U.S. NIS, indicating that our sample is representative. This should be comforting to clinicians who might be worried that our institution does not have a representative population, since the Mayo Clinic provides THA to local residents and is also a referral center. To our knowledge, no systematic changes occurred in referral patterns from primary care physicians to orthopedic surgery or policy for reimbursement for primary THA (except slight uniform reduction in compensation across the health care system) in the U.S. during the study period that would explain the time-trends in these characteristics. Another contribution from our study to the literature was our ability to provide overall and age-specific time-trends in BMI and to examine time-trends in extreme obesity.

An important finding from our study was the doubling of the rates of extreme obesity in 50- < 65 and 65- < 80 age groups and of depression in all 4 age groups. To our knowledge this is the first study to examine these important patient characteristics in various age groups, over time. Examination of these characteristics (extreme obesity, depression etc.) by age groups provides a detailed knowledge of their variation over time by age in primary THA patients. This knowledge can also allow policy makers to direct their efforts towards the group that is most at risk due to rapidly increasing prevalence of these clinical features that are associated with poor prognosis and worse outcomes after primary THA [12, 15, 18, 19, 36].

We also found that the prevalence of RA/inflammatory arthritis reduced by 65-75% in <50 and 65- < 80 age groups over the study period. This finding adds to the growing evidence that fewer patients with RA/inflammatory arthritis are requiring THA, compared to 1–2 decades ago [5, 37, 38]. This may at least partially be due to the availability and more frequent use of effective disease-modifying treatments for RA, including methotrexate and/or biologics [39, 40], as recommended in the RA treatment guidelines [41, 42].

The time-trends in the prevalence of extreme obesity, depression and anxiety were mostly independent of age, gender and medical comorbidity, as demonstrated in the multivariable-adjusted models. Anxiety and depression are well-recognized predictors of poorer pain and function outcomes after arthroplasty [14, 43–45]. This is an important finding, since there were significant time trends of decreasing patient age and increasing medical comorbidity over the same time-period. We noted minimal attenuation of odds ratios comparing unadjusted to multivariable-adjusted models, implying that increasing obesity, depression and anxiety in primary THA patients is not the result of increasing medical comorbidity or decreasing age.

In our study, we found rapidly increasing prevalence of moderate to severe renal disease, cerebrovascular disease and dementia over time. To our knowledge, this has not been reported previously in primary THA patients. This finding indicates that surgeons and patients need to be aware of increasing rate of certain comorbidities over time, which may be associated with an increase in peri-operative complications. This needs further study.

Our study confirms several previous findings including a reduction in mean age and increase in Deyo-Charlson index for patients undergoing primary THA. Findings from the study of the U.S. NIS showed that Deyo-Charlson index increased 0.42 to 0.55 (30% increase) from 1998–2008 in patients undergoing THA [9], similar to the 36% increase noted in our study. The magnitude of increases is lower compared to a 100% increase of comorbidity from 1 to 2 in Medicare sample from 1991 to 2008 [6]. This is not surprising due to the inclusion of all-comers in our and the NIS study vs. only patients 65 years and older (with higher comorbidity) in the Medicare sample. Our study also confirms a doubling of the prevalence of diabetes in patients undergoing primary THA over last 10–15 years, as reported in analyses of Medicare (7.1% in 1991–92 to 15.5% in 2007–08) [6] and the NIS data (6.8% in 1990–94 to 11% in 2000–04) [10].

Our study showed that the change in primary THA patient characteristics with increasing obesity and medical and psychological comorbidity is evident. These time-trends need to be accounted in research studies of THA outcomes and when health care plans and policy makers are making the reimbursement and coverage decisions. Data on several important patient characteristics are limited or absent in Medicare and NIS datasets that can be examined more comprehensively using data from total joint registries, similar to ours.

Our study has several limitations that should be taken into account while interpreting study findings. Our cohort study is subject to residual confounding due to study design, despite the adjustment for several important covariates in our multivariable-adjusted analyses. We used ICD-9 diagnostic codes to define anxiety and depression, and therefore these are likely under-reported. In addition, the increasing incidence of anxiety and depression over time might indicate either a better capture/reporting of these or a true increase in its incidence in this patient population. We are unable to distinguish between these two possibilities.

Study strengths include the use of height/weight data for BMI, a representative sample from a well-established U.S. total joint registry, availability of electronically captured data for more than a decade and robustness of our findings across several sensitivity models (age-adjusted vs. age- and sex-adjusted vs. age, sex- and comorbidity-adjusted models). The similarity of our cohort to other published studies of THA [8, 12, 13, 46] as well as the NIS sample [9, 47], supports the generalizability of these findings.

## Conclusion

In conclusion, we observed an increasing prevalence of obesity, medical and psychological comorbidity and decreasing prevalence of RA/inflammatory arthritis over a 13-year study period in patients undergoing primary THA. The time-trends differed by the age category. These time-trends in patient characteristics should be accounted for when comparing results over time and performing studies that include several years of data. Our study findings should be resource for public policy makers and those comparing resource use across interventions and procedures and making reimbursement decisions. These data represent the changing complexity of primary THA patients in the U.S., which can potentially impact outcomes.
